# Exosomes and STUB1/CHIP cooperate to maintain intracellular proteostasis

**DOI:** 10.1371/journal.pone.0223790

**Published:** 2019-10-15

**Authors:** Joao Vasco Ferreira, Ana Rosa Soares, José S. Ramalho, Teresa Ribeiro-Rodrigues, Catarina Máximo, Mónica Zuzarte, Henrique Girão, Paulo Pereira

**Affiliations:** 1 CEDOC, Chronic Diseases Research Centre, NOVA Medical School|Faculdade de Ciências Médicas, Universidade NOVA de Lisboa, Campo dos Mártires da Pátria, Lisboa, Portugal; 2 Coimbra Institute for Clinical and Biomedical Research (iCBR), Faculty of Medicine, University of Coimbra, Azinhaga de Santa Comba, Coimbra, Portugal; University of California, Davis, UNITED STATES

## Abstract

Deregulation of proteostasis is a main feature of many age-related diseases, often leading to the accumulation of toxic oligomers and insoluble protein aggregates that accumulate intracellularly or in the extracellular space. To understand the mechanisms whereby toxic or otherwise unwanted proteins are secreted to the extracellular space, we inactivated the quality-control and proteostasis regulator ubiquitin ligase STUB1/CHIP. Data indicated that STUB1 deficiency leads both to the intracellular accumulation of protein aggregates and to an increase in the secretion of small extracellular vesicles (sEVs), including exosomes. Secreted sEVs are enriched in ubiquitinated and/or undegraded proteins and protein oligomers. Data also indicates that oxidative stress induces an increase in the release of sEVs in cells depleted from STUB1. Overall, the results presented here suggest that cells use exosomes to dispose of damaged and/or undegraded proteins as a means to reduce intracellular accumulation of proteotoxic material.

## Introduction

Age-related diseases are complex and multifactorial. However it is becoming apparent that many of such diseases share common traits related to the deregulation of proteostasis [1]. Proteostasis refers to the maintenance of protein homeostasis and involves protein synthesis, folding and degradation [2]. The proteostasis network consists of molecular chaperones, the ubiquitin proteasome-system (UPS) and the autophagic/lysosomal system and often undergoes an age-related loss of function [3–5], leading to the intracellular and extracellular accumulation of undegraded, or otherwise toxic, material. Accumulation of proteotoxic material is associated with the onset of a variety of age-related diseases including Alzheimer´s [1], Parkinson´s [6], Huntington’s Disease [6], as well as, Age-related Macular Degeneration (AMD) [7, 8]. One of the most striking features of AMD is the age-dependent accumulation of extracellular deposits called drusens. Drusens are present in the earliest stages of AMD and are a major risk factor for vision loss [7]. While the pathophysiologic biogenesis of drusens remains unclear data suggests that these deposits contain undigested material released from the retinal pigmented epithelium (RPE) [8]. The RPE consists of a monolayer of post-mitotic cells and is responsible for the phagocytosis and clearance of the distal part of the light-sensitive photoreceptor outer segments.

Extracellular accumulation of protein oligomers or protein aggregates, is a hallmark of proteinopathies associated with ageing and disease [9] and implies the existence of mechanisms for the secretion of toxic proteins. Increasing evidence shows that many proteins, including beta-amyloid peptide, tau, alpha-synuclein, polyglutamine-expanded huntingtin (polyQ-htt), 43kDa TAR DNA-binding protein (TDP-43), superoxide dismutase (SOD1) and alphaB-crystallin are all secreted by cells in a way that may either alleviate intracellular proteostasis networks or lead to propagation of the disease by a “prion-like” mechanism [10–12].

A subgroup of extracellular vesicles, referred to as exosomes, were shown to be involved in the release of toxic protein oligomers [13–15]. Exosomes originate from multivesicular endosomes (MVEs) that via invagination of their limiting membrane form small intraluminal vesicles (ILVs) [16]. MVEs can fuse with lysosomes, leading to degradation of their content, or with the plasma membrane, leading to the release of exosomes [16]. Nonetheless, there is only very limited information on the molecular mechanisms linking intracellular accumulation of proteotoxic material and exosome-mediated disposal of undegraded proteins. Here, we provide evidence to suggest that the ubiquitin ligase STUB1 provides a link between these two processes.

STUB1 is a unique ubiquitin ligase containing three tetratricopeptide repeats (TPR domains) at its N-terminal and an U-box domain at its C-terminal. The U-box domain plays a key role in targeting proteins for ubiquitination [17, 18] while the TPR domain mediates the interaction with major cytoplasmic chaperones such as HSC/HSP70 and HSP90 [19, 20]. Through the action of both domains, STUB1 has a pivotal role in mediating triage decisions between protein folding and degradation [21–27]. Consistently, it has been shown that the activity of STUB1 promotes protein ubiquitination and degradation, while the loss of STUB1 activity leads to protein aggregation [25, 28]. Another important feature of STUB1 is its ability to regulate the synthesis of molecular chaperones by activating the transcription factor Heat Shock Factor 1 (HSF1), thus providing an additional level of control of proteostasis [29, 30].

In this study we use STUB1 inactivation to disrupt proteostasis in a human retinal pigment epithelium (ARPE-19) cell line. We show that inactivation of STUB1, results in protein aggregation and increased release of small extracellular vesicles (sEVs), including exosomes. Significantly, these sEVs are loaded with ubiquitinated and/or undegraded and oligomerized proteins. Our data further indicates that the loading of these proteins into sEVs, occurs at the level of MVEs and is dependent on the exosome secretion regulator Rab27 [31]. The secretion of these proteins, likely through exosomes, appears to be negatively regulated by HSF1 activation.

Overall, our data indicates that upon oxidative damage, cells use exosomes to dispose of oligomerized proteins, in a mechanism dependent on STUB1 and HSF1. This mechanism is likely to alleviate intracellular proteotoxic stress in RPE cells, as well as in other post-mitotic cells where accumulation of proteotoxic material contributes to disease.

## Material and methods

### Cell culture and treatments

The retinal pigment epithelium cell line ARPE-19 (LGC Promochem) was cultured in DMEM (Dulbecco’s modified Eagle’s medium) (1:1) supplemented with 10% fetal bovine serum (FBS), Penicillin/Streptomycin (100 U/ml:100 μg/ml) and 1% GlutaMax. Cells were cultured at 37°C under 5% CO2. When appropriate, cells were treated with the following agents: 20 μM MG-132 or Z-LLL-CHO (Calbiochem, 133407-82-86), 50nM Bafilomycon A1 (Millipore, 88899-55-2), 40mU/mL Glucose Oxidase (Sigma-Aldrich, G6125). Transient transfection of cells was performed with Jetprime (Polyplus-transfection, 114–07), according to manufacturer’s recommendations.

### Antibodies and reagents

The following antibodies were used: mouse anti-ubiquitin, dilution1:1000 (for Western Blot (WB)) 1:100 (for Immunofluorescence(IF))(Enzo, BML-PW0930-0100); goat anti-HIF1A, dilution of 1:1000 (Sicgen, AB0112-200); goat anti-MutYH, dilution 1:1000 (Sicgen, AB0118-200); goat anti-p53 dilution 1:1000 (Sicgen, AB0065-200); rabbit anti-IkB-a dilution 1:1000(Cell Signaling, 9242); goat anti-myc dilution 1:1000 (WB) 1:100 (IF) (Sicgen, AB0127-200); goat anti-Tubulin dilution 1:2000 (Sicgen, AB0046-200); goat anti-GAPDH dilution 1:2000 (Sicgen, AB0049-200); goat anti-Rab27 dilution 1:500 (Sicgen, AB7223-200); mouse anti-p62 dilution 1:500 (WB) (Santa Cruz, sc-28359); rabbit anti-p62 dilution 1:100 (IF)(Cell Signaling, 5114S); rabbit anti-CHIP dilution 1:100 (IF) (Cell Signaling, 05/2013); goat anti STUB1 dilution 1:500 (abcam, ab2482); goat anti-CD63 dilution 1:1000 (Sicgen, AB0047-200); rabbit anti-Oligomer (A11) dilution 1:1000 (Invitrogen, AHB0052); mouse anti-HSF1 dilution 1:500 (WB) and 1:100 (IF)(Santa Cruz, SC-17757); rat anti-Hsc70 clone 1B5, dilution of 1:1000 (Stressgen, ADI-SPA-815); goat anti-Calnexin, dilution 1:1000 (Sicgen, AB0041-200); goat anti-Na^+^/K^+^ ATPase, dilution 1:1000 (Sicgen, AB0306-100); mouse anti-Hsp70, dilution 1:1000 (Enzo, ADI-SPA-810); rabbit anti-Hsp40 dilution 1:1000 (Enzo, ADI-SPA-400); and horseradish peroxidase-conjugated secondary goat anti-mouse (Bio-Rad, 170–6516), goat anti-rabbit (Bio-Rad, 170–6515), rabbit anti-goat (Bio-Rad, 172–1034) and goat anti-rat (Invitrogen, A10549) dilution of 1:5000. Alexa Fluor 568-conjugated goat anti-mouse (Invitrogen, A-11019), Alexa Fluor 488-conjugated goat anti-rabbit (Invitrogen, A-11008), Alexa Fluor 488-conjugated goat anti-mouse (Invitrogen, A-11001), Alexa Fluor 568-conjugated goat anti-rabbit (Invitrogen, A-11011), Alexa Fluor 633-conjugated donkey anti-goat (Invitrogen, A-21082), dilution of 1:250. Egg Liss Rhod PE (L-α-Phosphatidylethanolamine-N-(lissamine rhodamine B sulfonyl) (Ammonium Salt) (Egg-Transphosphatidylated, Chicken), powder) (Avanti Polaris, 810146P). DAPI (4',6-Diamidino-2-Phenylindole, Dihydrochloride) (Invitrogen, D1306). Protein G–Sepharose (GE Healthcare, 17-0618-01). Nitrocellulose membranes (GE Healthcare, 88018). ECL (GE Healthcare, RPN1235). Optiprep iodixanol density media (Sigma-Aldrich, D1556). ONE-Glo^™^ Luciferase Assay System (Promega, E6110).

### Plasmids

For this work we used the following plasmids: pcDNA3.1 c-myc-STUB1 wt, pcDNA3.1 c-myc-STUB1 K30A, pcDNA3.1 c-myc-STUB1H260Q [32]. pcDNA3.1 hHsf-1-c-myc 68 [33]. pGL4.23 HSE wt (x4)-Luciferase [34].

### Viral shRNA production and infection

For Rab27 Knock-down, Rab27a and Rab27b micro-RNA (miRNA)-expressing vectors were constructed by inserting an inverted repeat of Rab27-specific 21-nucleotide sequences into pcDNA6.2-GW/EmGFP-miR plasmid harboring PolII promoter obtained from Thermo Scientific. These sequences are fused with GFP coding sequence. For miRNA targeting of human Rab27a and Rab27b, the synthesized oligonucleotides were annealed and ligated into pcDNA6.2-GW/EmGFP-miR according to manufacturers’ instructions. The oligonucleotides used are in Table 1 (underline indicates RNA interference target sequences). All plasmids were verified by DNA sequencing and tested for the Knock-down (unpublished data). The four miRNA were cloned in tandem into one pcDNA6.2-GW/EmGFP-miR plasmid according to manufacturers’ instructions. Tandem miRNA sequences were transferred into pAd adenoviral vector from Thermo Scientific using Gateway technology.

**Table 1:** RNA interference target sequences.

Rab 27a	miR1	5’—TGCTGAAACTTTGCTCATTTGTCAGGGTTTTGGCCACTGACTGACCCTGACAAGAGCAAAGTTT—3’
		3’—CCTGAAACTTTGCTCTTGTCAGGGTCAGTCAGTGGCCAAAACCCTGACAAATGAGCAAAGTTTC—5’
	miR2	5’—TGCTGTTAACTGATCCGTAGAGGCATGTTTTGGCCACTGACTGACATGCCTCTGGATCAGTTAA—3’
		3’—CCTGTTAACTGATCCAGAGGCATGTCAGTCAGTGGCCAAAACATGCCTCTACGGATCAGTTAAC—5
Rab 27b	miR3	5’—TGCTGATTGACTTCCCTCTGATCTGGGTTTTGGCCACTGACTGACCCAGATCAGGGAAGTCAAT—3’
		3’—CCTGATTGACTTCCCTGATCTGGGTCAGTCAGTGGCCAAAACCCAGATCAGAGGGAAGTCAATC—5
	miR4	5’—TGCTGTTTCCCTGAAGATCCATTCGGGTTTTGGCCACTGACTGACCCGAATGGCTTCAGGGAAA—3’
		3’—CCTGTTTCCCTGAAGCCATTCGGGTCAGTCAGTGGCCAAAACCCGAATGGATCTTCAGGGAAAC—5

For shRNA targeting of human STUB1 and miRNA targeting of human Rab27a/b, viral particles were produced and cells were infected as described before [35].

### Dot-blot filter retardation assay

Cells were collected from dishes with ice cold PBS using a cell scrapper and centrifuged at 15000g for 10 minutes. Cells were resuspended in buffer (50mM TRIS-HCl pH 7.4, 150mM NaCl, 10mM iodoacetamide, 2mM PMSF, protease inhibitor cocktail) and lysed by sonication (4 rounds of 10 seconds at 4°C). Lysates were centrifuged at 15000g for 5 min at 4°C and the resulting pellets were resuspended in buffer. 20 ug of protein of each sample was incubated in buffer with 0.5 to 2% SDS or 2% SDS at 95°C. After gentle rocking for 2h, samples were filtered on a BioRad dot-blot filtration unit through a nitrocellulose membrane. The membranes were blocked with 5% non-fat milk in TBS-T (Tris-buffered saline, 0.1% Tween 20) and probed for the proteins of interest.

### Exosomes/small extracellular vesicles (sEVs) and microvesicles (MVs) isolation

EVs derived from 30 million cultured cells were isolated from 20 mL conditioned medium over a period of 24h. Exosome-depleted medium was prepared accordingly to Lässer and collegues [36]. After incubation, the medium was collected and sEVs and MVs were isolated by ultracentrifugation, as described before [36]. Briefly, the harvested supernatant was subjected to differential centrifugation at 4°C, starting with a centrifugation at 300g, for 10 min followed by a centrifugation at 16,500g, for 20 min. From this pellet, the MVs fraction was collected. To remove larger particles, the supernatant was filtered with a 0.22μm filter unit, after which it was ultracentrifuged at 120,000g, for 70 min. The resulting pellet was washed with PBS, and after ultracentrifugation, sEVs were resuspended in PBS. MVs and sEVs pellets were denatured with SDS sample buffer containing reducing agents (Laemmli buffer). Samples were then heated at 95°C, for 5 min, separated by SDS-PAGE, transferred to nitrocellulose membranes, and probed with appropriate antibodies. The same experimental approached was used in cell lysates, as applicable. We have submitted all relevant experimental data to the EV-TRACK knowledgebase (EV-TRACK ID: EV190055) [37].

### Separation of sEVs on discontinuos sucrose gradient

Exosome-enriched pellets were placed at the bottom of an ultracentrifuge tube, filled with a discontinuous gradient of sucrose (from 2.5M to 0.4M) and ultracentrifuged overnight at 210,000g. Sequential fractions were collected (1mL each, from the top to the bottom of the tube) and each fraction was weighed to determine its average density. Fractions were collected by ultracentrifugation at 120,000g for 70min. Samples were sonicated 3x and filtered on a BioRad dot-blot filtration unit through a nitrocellulose membrane. The membranes were blocked with 5% non-fat milk in TBS-T and probed for the proteins of interest.

### Immunoprecitation

Cells were collected from dishes with ice cold PBS using a cell scrapper and centrifuged at 15000g for 10 minutes. In all cases pellets were resuspended in 150μl of lysis buffer (50mM TRIS-HCl pH 7.4, 150mM NaCl, 10mM iodoacetamide, 2mM PMSF, protease inhibitor cocktail and 0.5% of NP-40) and sonicated 3 times, 1s each at 4°C. After, samples were centrifuged at 15000g for 10minutes and pellets were discarded. All samples were incubated with 2μg of the antibody against the protein of interest overnight at 4°C. Subsequently 30μl of protein G–Sepharose was added to the sample and incubations proceeded at 4°C for 2h. Beads were washed 3 times with lysis buffer containing 0.15% of NP-40, denatured with 1× Laemmli buffer and boiled at 100°C. Samples were then analyzed by SDS-PAGE. The membranes were blocked with 5% non-fat milk in TBS-T and probed for the proteins of interest.

### Cell loading with N-rhodamine-phosphatidylethanolamine (RhoB-PE)

Lipids were solubilized in absolute ethanol. The RhoB-PE ethanolic solution was injected with a Hamilton syringe into DMEM serum free media, in a final concentration of 2μM, and added to cells for 1h at 4°C. Cells were then washed 3 times with PBS and incubated with DMEM supplemented with 10% FBS, for 1h at 37°C after which cells were fixed with 4% Paraformaldehyde (PFA) for immunofluorescence [38]. For exosome analysis, cells were incubated with DMEM Exo-free media for 24h and after isolation by differential ultracentrifugation exosomes were quantified by measuring fluorescence using Tecan Infinite F200 PRO instrument.

### Immunofluorescence

Cells were fixed with 4% PFA for 10 min, washed three times with PBS, permeabilized for 10 min with 0.2% Triton X-100 and blocked for 20min with 1% BSA. Cells were incubated with primary antibodies for 1h, washed three times and incubated with secondary antibodies and DAPI for 1h. After three washes in PBS, coverslips were mounted MOWIOL 4–88 Reagent mounting medium and observed with a Zeiss LSM510 confocal microscope. Co-localization and vesicle size were evaluated using ImageJ software.

### Nanosight tracking analysis (NTA)

sEVs isolated from STUB1-DN mutant cells were subjected to Nanosight tracking analysis (NTA), using a NanoSight LM 10 instrument (NanoSight Ltd). Settings were optimized and kept constant between samples. Each video was analyzed to give mean, mode, median and estimated concentration for each particle size. Data were processed using NTA 2.2 analytical software.

### Reporter assay

HSF1 activity was measured by transducing cells with the reporter gene Luciferase under the control of the Heat Shock Element (HSE), using ONE-Glo^™^ Luciferase Assay System (Promega), according to the manufacturer’s specifications.

### Transmission electron microscopy (TEM)

sEVs and MVs were fixed with 2% paraformaldehyde (PFA) and deposited on Formvar-carbon coated grids. Samples were washed with PBS and fixed with 1% glutaraldehyde for 5 min. After washing with distilled water, grids were contrasted with uranyl-oxalate pH 7, for 5 min, and transferred to methyl-cellulose-uranyl acetate, for 10 min on ice. Observations were carried out using a Tecnai G2 Spirit BioTWIN electron microscope (FEI) at 80 kV.

### Statistical analysis

Data are reported as the means ± standard deviation of at least three independent experiments. Comparisons between multiple groups were performed by one-way analysis of variance test (ANOVA) with Tukey’s multiple comparison tests, using GraphPad Prism 5.0 software (GraphPad Software). For comparison between two groups, the paired t-test was used. In all cases, p < 0.05 was considered significant.

## Results

### Expression of STUB1 dominant-negative mutants in ARPE-19 cells leads to the accumulation of ubiquitinated material and the formation of insoluble protein aggregates

We, and others, have previously showed that the STUB1K30A and STUB1H260Q mutants act as dominant-negatives (DN) of wtSTUB1 [22, 23, 32]. Thus, we expressed the two STUB1 mutants: a mutant that does not have a functional TPR domain (STUB1K30A), and that, as a consequence, is unable to bind chaperones, and a second mutant that has an impaired ubiquitin ligase activity (STUB1H260Q). We anticipated that the expression of two different STUB1-DN mutants would impair cellular proteostasis, leading to the accumulation of undegraded proteins.

**Fig 1:**
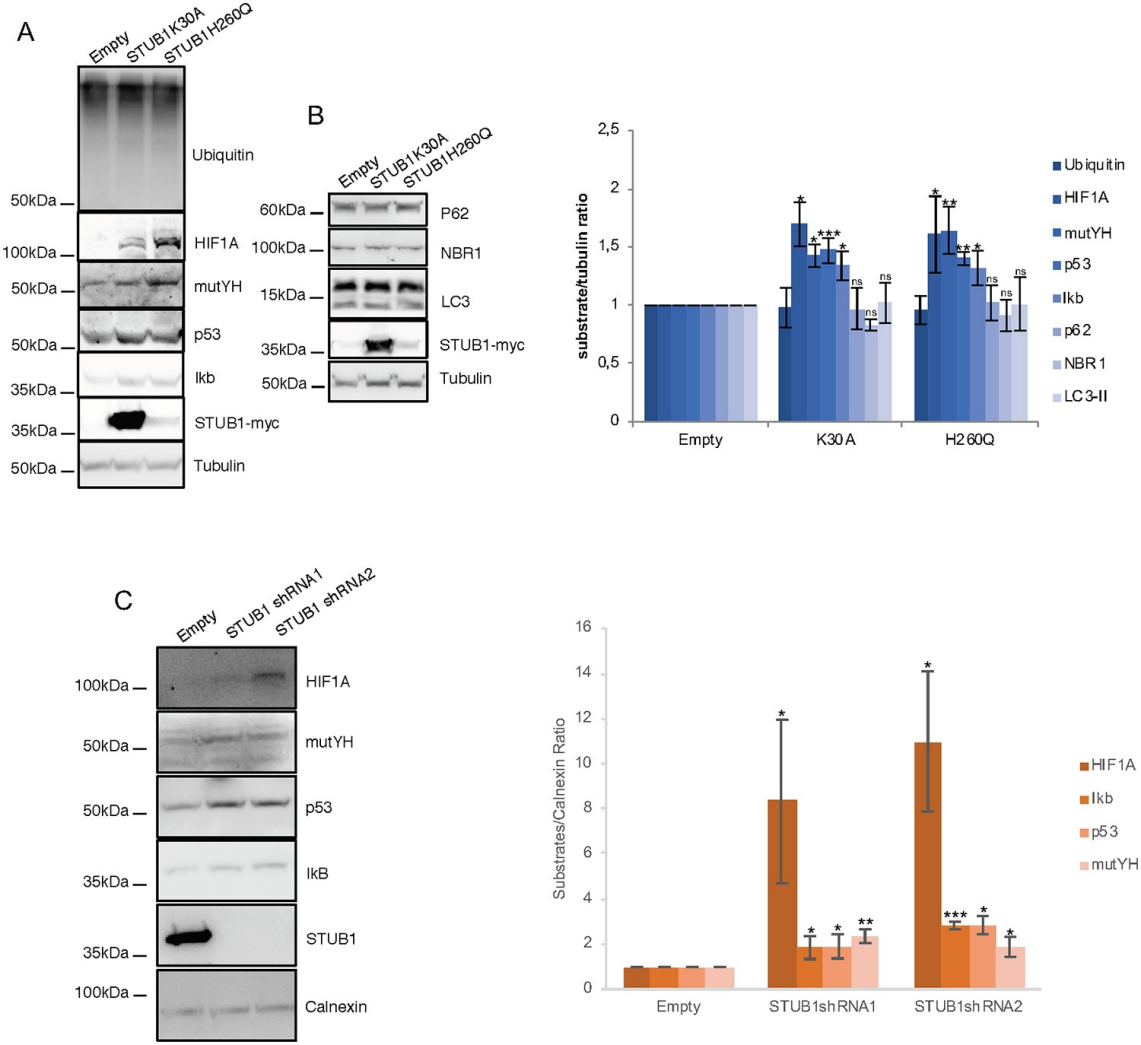
Expression of STUB1-DN mutants induces stabilization of proteasomal substrates. ARPE-19 cells were transduced using lentiviral particles containing vectors for the expression of either STUB1K30A or STUB1H260Q or with adenoviral particles containing shRNA against STUB1. Control cells were transduced with an empty vector. (A,B) Western blot of whole cell lysates with antibodies against: A) the proteasomal substrates HIF1A, mutYH, p53, IkB and B) the autophagic substrates LC3, p62, NBR1; ubiquitin, tubulin and myc tag. Expression of the STUB1 mutants increases the protein levels of proteasomal but not lysosomal substrates. C) Western blot of whole cell lysates with antibodies against STUB1 and the proteasomal substrates HIF1A, mutYH, p53, IkB. Depletion of STUB1 increases the protein levels of proteasomal substrates. All samples were analyzed under the same experimental conditions. The results represent the mean ±SD of N = 3 independent experiments (n.s. nonsignificant; *p < 0.05; **p < 0.01; ***p < 0.001).

We transduced ARPE-19 cells with the STUB1-DN mutants. After transduction, the protein expression levels of the STUB1K30A mutant are higher than the levels of the STUB1H260Q mutant. Nonetheless, data shows that stable expression of both STUB1-DN mutants leads to a similar increase in the levels of a number of UPS substrates (HIF1A, mutYH, p53 and IkB), while the overall levels of ubiquitin conjugates remains unchanged (Fig 1A). On the other hand, the expression of DN mutants fails to stabilize the autophagic/lysosomal substrates p62, NBR1 and LC3 (Fig 1B).

Subsequently we used adenoviral particles to transduce two different shRNAs against STUB1 in ARPE-19 cells. Results show that STUB1 depletion also leads to an increase in the protein levels of the UPS substrates (Fig 1C).

**Fig 2:**
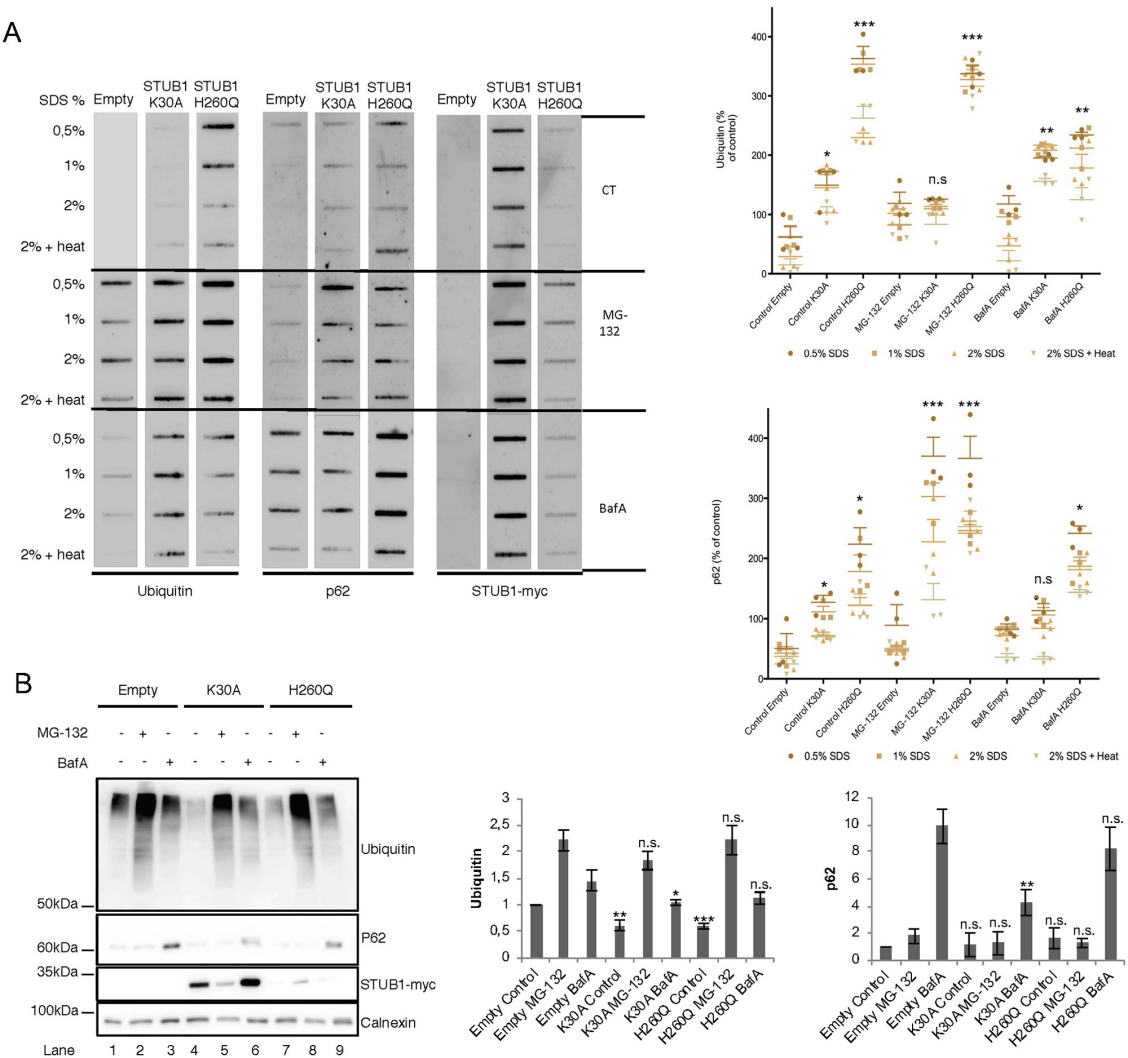
Expression of STUB1DN mutants leads to the formation of protein aggregates. ARPE-19 cells were transduced using lentiviral particles containing vectors for the expression of either STUB1K30A or STUB1H260Q. Control cells were transduced with empty vector. Cells were further incubated in the presence or absence of 10uM of MG-132 and 50nM of BafA for 6h. A) Cell extracts of proteins insoluble in increasing concentrations of SDS where filtered through a nitrocellulose membrane and trapped proteins were blotted with antibodies directed against ubiquitin, p62 and myc tag. Expression of STUB1-DN mutants increases the amount of protein aggregates trapped in the membrane both in the presence or absence of MG-132 or BafA. B) Western blotting for the proteins present in the supernatant (soluble proteins) shows a decrease in the levels of ubiquitin conjugates in the STUB1-DN mutant expressing cells (compare lane 1 with lane 4 and 7). All samples were analyzed under the same experimental conditions. The results represent the mean ±SD of N = 3 independent experiments (n.s. nonsignificant; *p < 0.05; **p < 0.01; ***p < 0.001).

To assess the formation of insoluble protein aggregates upon STUB1-DN mutant expression, protein pellets were incubated with increasing concentrations of SDS (0.5–2%) or 2% SDS followed by a 5 minutes incubation at 95°C. Subsequently, proteins were filtered through a membrane, and blotted with antibodies raised against protein aggregate markers ubiquitin and p62 [39]. Results in Fig 2A, revealed that both mutants increase the formation of protein aggregates positive for both markers. Interestingly, SDS-PAGE of the soluble proteins in the supernatant also shows that the levels of ubiquitin conjugates decrease in STUB1-DN mutant cells (Fig 2B, compare lane 1 with lanes 4 and 7).

We next inhibited the proteasome with MG-132 as a means to disrupt proteostasis. In these conditions, cells transduced with STUB1H260Q but not with STUB1K30A show an increase in ubiquitin positive protein aggregates (Fig 2A). On the other hand, cells expressing both STUB1H260Q and STUB1K30A, show higher levels of p62 positive aggregates when compared to the empty vector. Additionally, inhibition of the lysosome with Bafilomycin A1 (BafA) leads to an increase in the accumulation of ubiquitin positive aggregates in cells expressing both STUB1-DN mutants, while expression of STUB1H260Q, but not of STUB1K30A, leads to an accumulation of p62 positive aggregates (Fig 2A). Overall, data gathered from the filter trap assay indicates that the expression of STUB1-DN mutants in ARPE-19 cells leads to an increase in the formation of intracellular protein aggregates.

Consistently, confocal microscopy of ARPE-19 cells expressing STUB1-DN mutants, show the formation of puncta positive for ubiquitin and p62 (S1 Fig). The co-localization between ubiquitin and p62 increases from 0.61% (empty vector) to 1.04% for the STUB1K30A mutant and to 5.44% for STUB1H260Q mutant (S1 Fig).

Altogether, data indicates that inactivation of STUB1 mutants inhibits the degradation of proteins by the proteasome and induces the accumulation of protein aggregates positive for ubiquitin and p62.

### STUB1 inactivation enhances sEVs release

**Fig 3:**
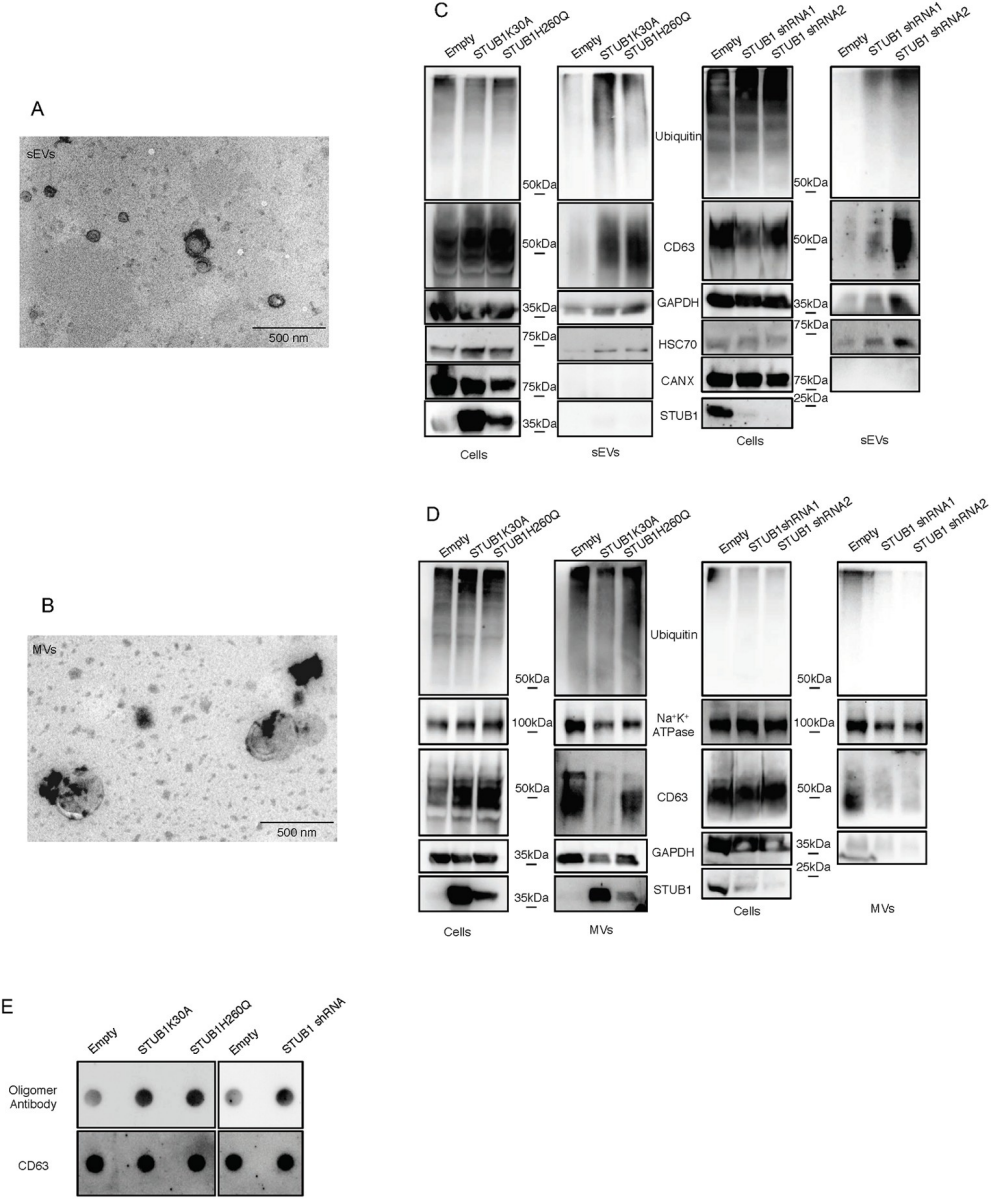
STUB1 inactivation stimulates the secretion of extracellular vesicles positive for sEVs markers. ARPE-19 cells were transduced using lentiviral particles containing vectors either for the expression of the STUB1K30A and STUB1H260Q mutants or with adenoviral particles containing shRNA against STUB1. Control cells were transduced with an empty vector. A,B) TEM images of sEVs (A) and MVs (B) isolated by sequential centrifugation. Size analysis of the vesicles shows that the 120,000g fraction contains particles with sizes smaller than 200nm (sEVs) and that the 16,500g fraction contains particles larger than 200nm (MVs). C,D) SDS-PAGE of cells extracts or vesicles isolated by sequential centrifugation from cell culture media were blotted with antibodies against ubiquitin, CD63, GAPDH, Hsc70, Calnexin (exosome negative marker [40, 41]), Na^+^K^+^ ATPase and myc tag or STUB1. STUB1-DN mutants expression and STUB1 depletion increases the release of sEVs, but not of other MVs, loaded with ubiquitinated proteins. E) Dot-Blot of 10ug of isolated sEVs incubated with an antibody that recognizes protein oligomers (A11) and the sEVs marker CD63. Both the expression of STUB1-DN mutants and the depletion STUB1 increases the levels of protein oligomers in sEVs. All samples were analyzed under the same experimental conditions.

Extracellular vesicles can be divided into microvesicles (MVs) (1000–100 nanometers), formed by the shedding of the plasma membrane, and exosomes (100–30 nanometers), that originate from the fusion of multivesicular endosomes (MVEs) with the plasma membrane. There is still a lack of consensus on specific markers for exosomes and the most common method for exosome isolation, ultracentrifugation, is unable to separate exosomes from other populations of small extracellular vesicles such as ectosomes [40]. We separated MVs from sEVs according to their size by differential ultracentrifugation and filtration of the media of ARPE-19 cells expressing the STUB1-DN mutants. Isolated vesicles of >200nm are refered to as microvesicles (MVs), while vesicles smaller than 200nm (that include exosomes) are called sEVs. We confirmed the size of the secreted vesicles by TEM (Fig 3A and 3B). Western blotting of 2.5% of whole cell lysates and vesicle samples (100% of isolated vesicles) shows an increase in the levels of ubiquitin conjugates, as well as the extracellular vesicles markers CD63, GAPDH and Hsc70 [40, 41], in the sEVs samples from STUB1-DN expressing cells (Fig 3C). As a negative marker for exosomes, we used calnexin [40, 41]. On the other hand MVs fractions, labeled with CD63, GAPDH and the MVs specific marker Na^+^K^+^ ATPase [42], shows no increase in the levels of ubiquitin conjugates or MVs markers (Fig 3D). We next depleted STUB1 from ARPE-19 cells using adenoviral vectors for two different shRNAs of STUB1. Data in Fig 3C shows an increase in the levels of ubiquitinated proteins and sEVs markers. On the other hand, MVs exhibit decreased levels of these proteins following STUB1 depletion (Fig 3D). Overall, the expression of STUB1-DN mutants or STUB1 depletion, results in an increase in sEVs, but not MVs release. Furthermore, we analyzed the sEVs fractions using the same amount of protein by dot-blot. Labeling of the dot-blot with an antibody that recognizes protein oligomers [43] (S2A and S2B Fig), indicates that both STUB1-DN mutants expression and STUB1 depletion leads to an increase in the levels of protein oligomers present in sEVs (Fig 3E). A sucrose density gradient to separate sEVs from non-membranous materials, such as, protein aggregates (which do not float in sucrose), shows that oligomerized proteins are present in sEVs fractions, with characteristic exosomal densities (S2C Fig). Interestingly, a subpopulation of sEVs derived from STUB1-DN mutant expressing cells display a lower floatation profile, indicating that oligomer loading increases the density of, if not all, some of the secreted sEVs.

Next, we assessed sEVs release after proteolysis impairment using proteasome (MG-132) or lysosome (BafA) inhibition, in cells expressing STUB1-DN mutants. Proteasome inhibition induces a mild increase in sEVs release, while lysosomal inhibition leads to a much higher and significant increase in sEVs release in all conditions (S3 Fig). This observation is consistent with previous reports indicating that lysosome inhibitors strongly stimulate the release of exosomes [44].

**Fig 4:**
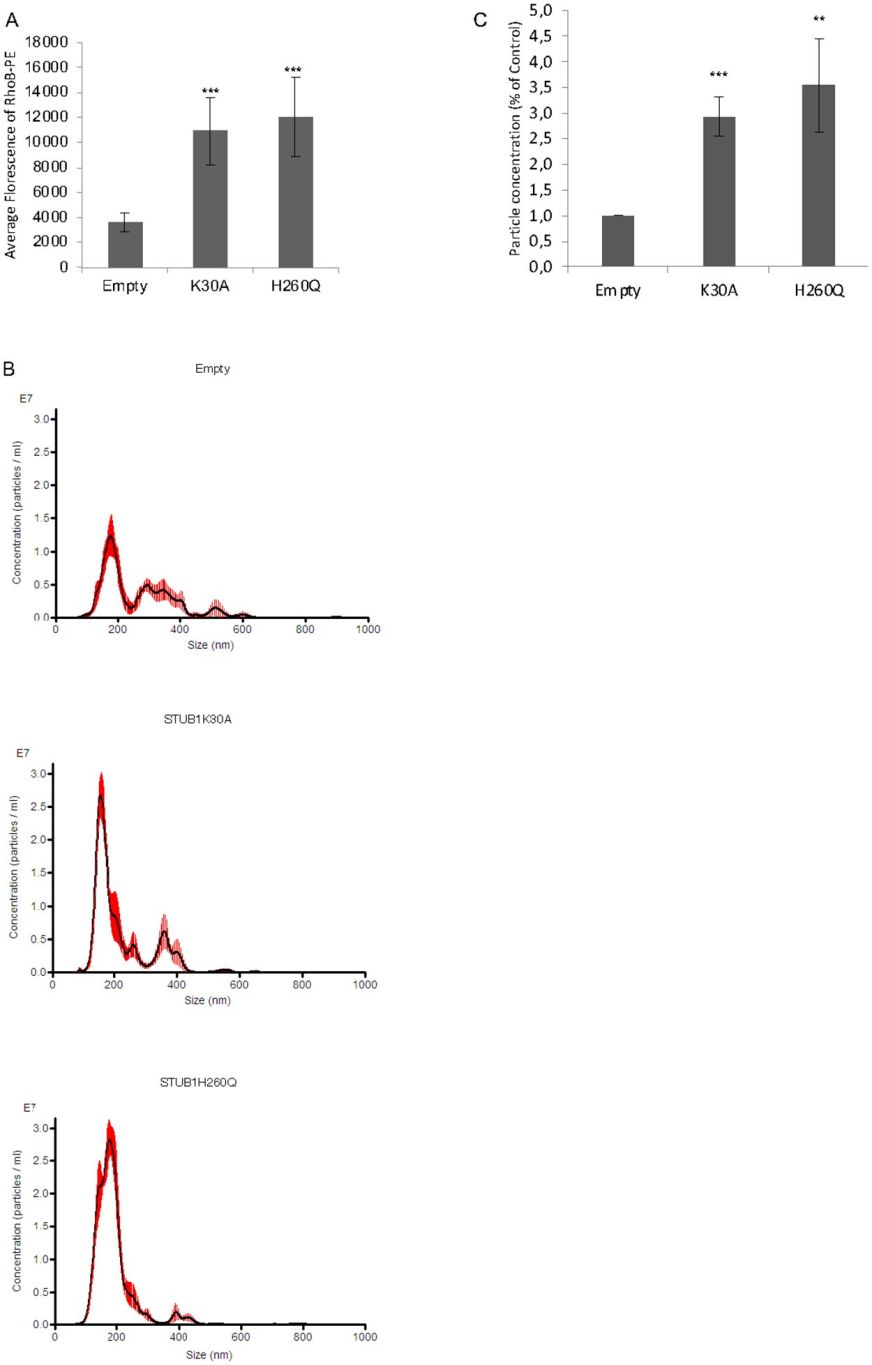
STUB1 DN-mutants expression stimulates sEVs secretion. ARPE-19 cells were transduced using lentiviral particles containing vectors either for the expression of the STUB1K30A and STUB1H260Q mutants. A) Cells were incubated with the MVE/exosome marker dye RhoB-PE prior to exosome isolation. Fluorescence quantification of the isolated exosomes shows an increase in cells expressing STUB1-DN mutants. B) Particle counting using nanoparticle tracking system (NanoSight). C) STUB1-DN mutants expression increases the number of exosomes released by ARPE-19 cells. All samples were analyzed under the same experimental conditions. The results represent the mean ±SD of N = 3 independent experiments (n.s. nonsignificant; *p < 0.05; **p < 0.01; ***p < 0.001).

The release of sEVs by ARPE-19 cells was further assessed by two alternative methods. sEVs isolated from cell culture media supernatants were quantified by laser scattering using NanoSight or by incubating ARPE-19 cells with Phosphatidylethanolamine (PE) conjugated to Lissamine-RhodamineB [45]. The incubation of cells with Lissamine-RhodamineB-PE allows for its integration in the plasma membrane and subsequent internalization. Upon internalization, PE is not recycled and follows the endocytic pathway, such that the only fluorescence retrieved from cell media originates from released exosomes. Both Lissamine-RhodamineB-PE (RhoB-PE) fluorescence measurement and particle counting using NanoSight show that sEVs release is increased in cells expressing STUB1-DN mutants (Fig 4A–4C).

**Fig 5:**
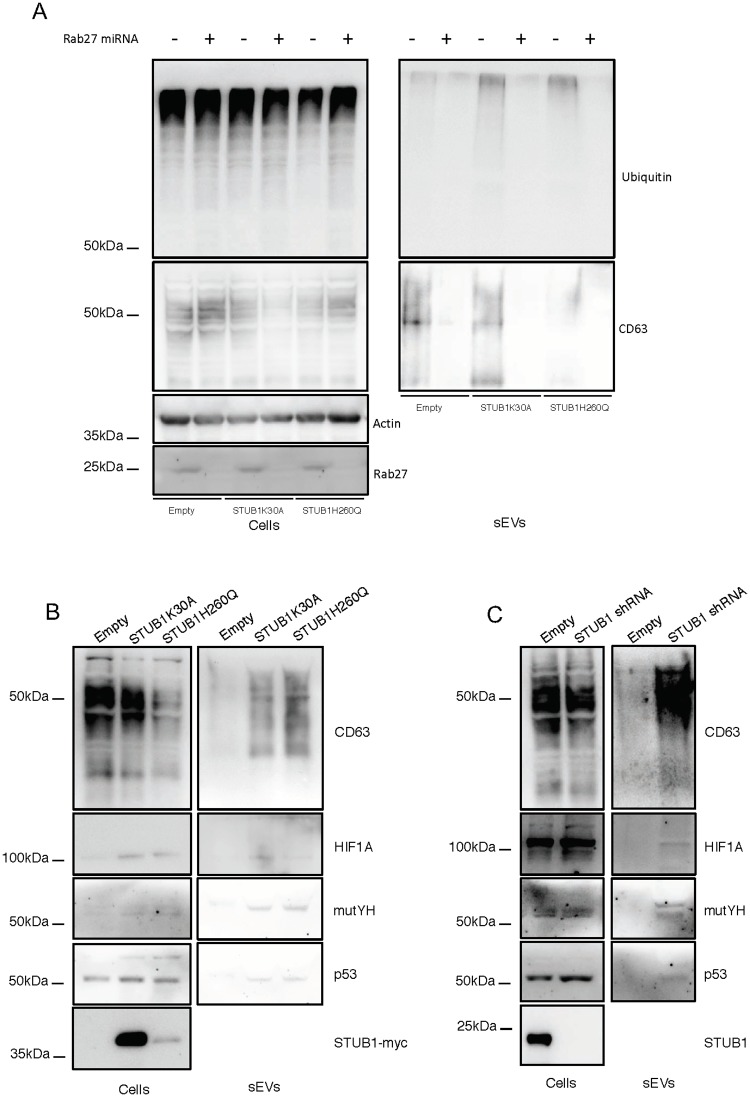
Rab27 depletion prevents the secretion of ubiquitinated proteins by sEVs and STUB1 inactivation leads to an increase of proteasomal substrates in secreted sEVs. ARPE-19 cells were transduced using lentiviral particles containing vectors for the expression of either STUB1K30A or STUB1H260Q, with adenoviral particles containing shRNA against STUB1 or with adenoviral particles containing miRNA against Rab27. Control cells were transduced with an empty vector. A) Western blot of whole cell lysates and sEVs samples with antibodies against ubiquitin, CD63 and GAPDH shows that depletion of Rab27 prevents the secretion of vesicles loaded with ubiquitinated proteins. B,C) Western blot of whole cell lysates and sEVs sample with antibodies against CD63, HIF1A, mutYH and p53. The expression of STUB1-DN mutants, as well as, the depletion of STUB1 increases the presence of proteasomal substrates in released sEVs. All samples were analyzed under the same experimental conditions.

To further confirm the localization of the ubiquitinated cargo in exosomes we depleted Rab27 to inhibit exosome secretion [31]. Data shows that inhibition of exosomal release, measured by the exosomal marker CD63, as well as, by NTA is followed by a decrease both in the in the levels of ubiquitin conjugates (Fig 5A) and the number of secreted particles of sizes <200nm (S4A Fig). Moreover, to monitor for the presence of specific UPS substrates in secreted sEVs, we blotted isolated fractions with antibodies raised against p53, mutYH and HIF1A. Data shows that STUB1-DN mutants expression, as well as, STUB1 depletion increases the presence of these substrates in sEVs (Fig 5B and 5C). Moreover, the depletion of Rab27 inhibited the secretion of the proteasomal substrates in the isolated fractions (S4B and S4C Fig), indicating that exosomes are likely to carry these proteins into the extracellular space.

**Fig 6:**
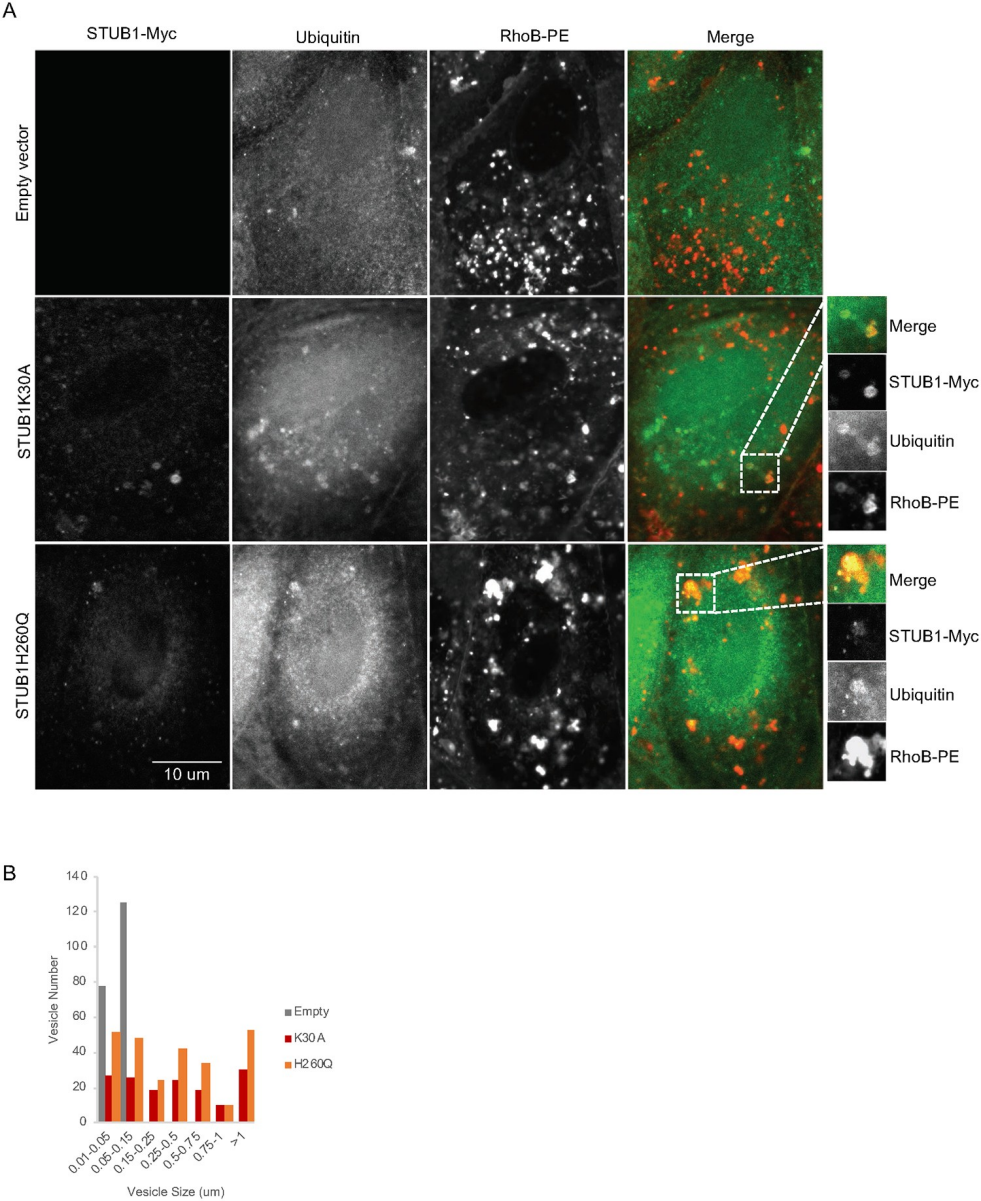
Ubiquitin colocalizes with MVEs in cells expressing STUB1-DN mutants. ARPE-19 cells were transduced using lentiviral particles containing vectors for the expression of either STUB1K30A or STUB1H260Q. Control cells were transduced with empty vector. A) Immunofluorescence using confocal microscopy with antibodies against myc, ubiquitin and the RhoB-PE dye for MVE labeling shows an increase in puncta positive for ubiquitin, STUB1-DN mutants and RhoB-PE. B) Quantification of size and number of vesicles labelled with RhoB-PE dye shows an increase in the frequency of larger vesicles in the STUB1-DN mutants.

Exosomes originate from the fusion of MVEs with the plasma membrane, thus we anticipate that the loading of undegraded proteins occurs during formation of MVEs. To address this hypothesis, MVEs were labeled with the fluorescent lipid RhoB-PE [45]. Confocal microscopy shows a significant increase in the colocalization between RhoB-PE and ubiquitin in cells expressing STUB1-DN mutants (Fig 6A and S5 Fig) or in cells depleted of STUB1 (S6A Fig). Also, RhoB-PE labeled vesicles are enlarged when STUB1 is inactivated (Fig 6B and S6B Fig). Additionally, these expanded vesicles contain STUB1-DN mutants (Fig 6A).

Overall, data indicates that STUB1 inactivation leads to the formation of insoluble aggregates, as well as, to an increase in the secretion of sEVs, that are likely to be exosomes, loaded with ubiquitinated and/or oligomerized proteins.

### Oxidative stress induces protein aggregation and increases sEVs release

Accumulation of oxidized proteins is a hallmark of ageing [46] and reactive oxygen species have been reported to induce protein damage and exosome release [47].

**Fig 7:**
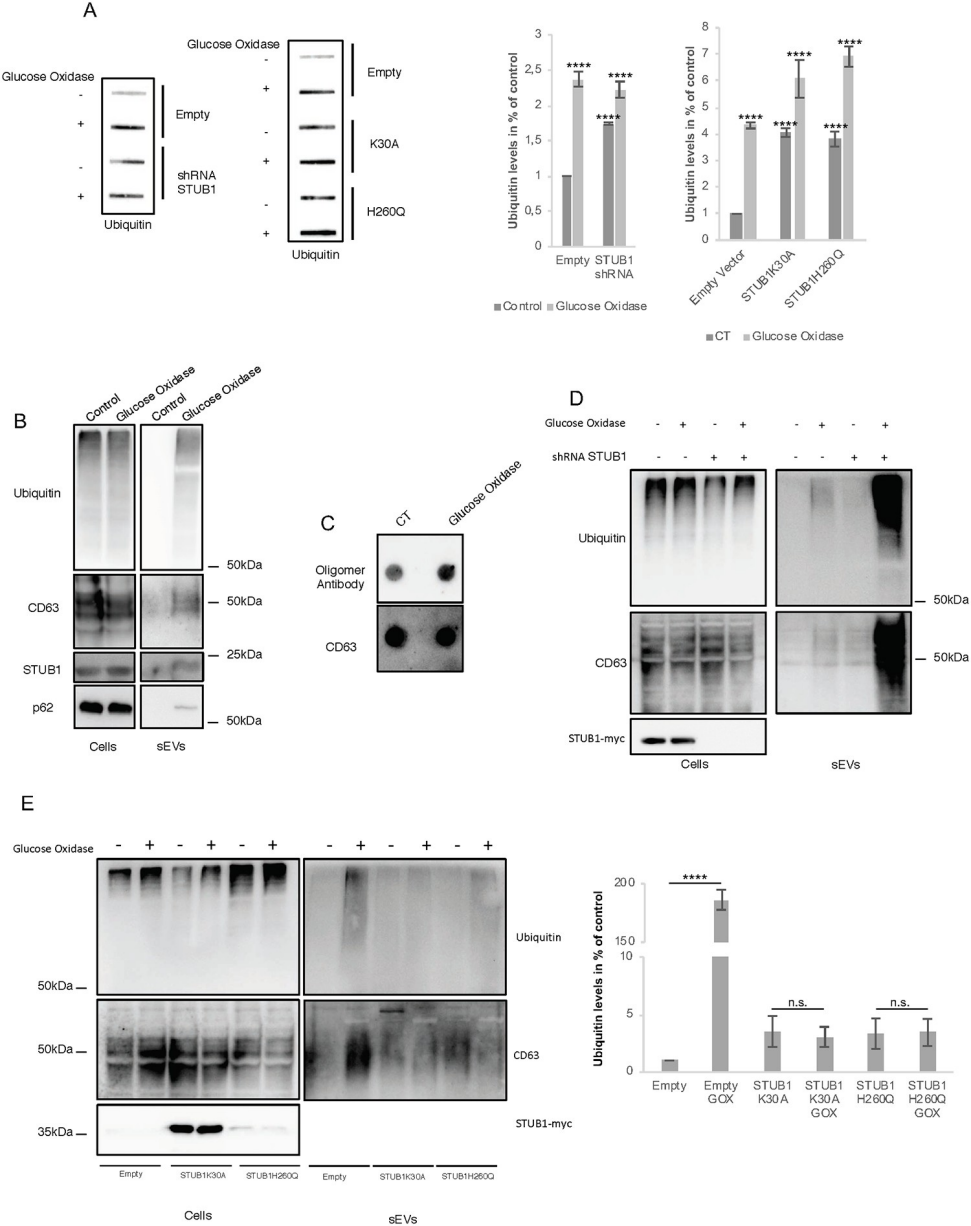
Oxidative stress stimulates sEVs secretion via a mechanism that is exacerbated upon STUB1 depletion but abrogated by the expression of STUB1-DN mutants. ARPE-19 cells were transduced with lentiviral particles containing vectors either for the expression of the STUB1K30A and H260Q mutants or with adenoviral particles containing shRNA against STUB1. Control cells were transduced with empty vector. Cells were maintained in the presence or absence or 40 mU of GOx for 12h. A) Cell extracts of proteins insoluble in 2% SDS at 95°C, were filtered through a nitrocellulose membrane and trapped proteins were blotted with antibodies against ubiquitin. GOx incubation increases the formation of insoluble protein aggregates in all conditions. (B,D,E) SDS-PAGE of cell extracts or sEVs isolated by sequential centrifugation from cell culture media were blotted with antibodies against ubiquitin, CD63, GAPDH, p62 or STUB1. B) GOx incubation leads to an increase in the release of sEVs loaded with ubiquitin as well as STUB1 and CD63. C) Dot-Blot of 10ug of isolated sEVs incubated with an antibody that recognizes protein oligomers and with an antibody for the sEVs marker CD63. GOx incubation increases the levels of protein oligomers. D) GOx incubation and STUB1 depletion have a cumulative effect in sEVs release. E) Expression of STUB1-DN mutants abrogates sEVs release in the presence of GOx. All samples were analyzed under the same experimental conditions. The results represent the mean ±SD of N = 3 independent experiments (n.s. nonsignificant; *p < 0.05; **p < 0.01; ***p < 0.001; ****p< 0.0001).

To assess the effects of oxidative damage in protein aggregation and sEVs release in ARPE-19 cells, we incubated cells with the enzyme glucose oxidase (GOx). GOx is an oxido-reductase that catalyzes the oxidation of glucose to hydrogen peroxide and D-glucono-δ-lactone, thus maintaining a steady production of hydrogen peroxide throughout the incubation time [48]. Incubation with GOx of ARPE-19 cells expressing either empty vector, STUB1 DN-mutants or depleted for STUB1, induces the formation of insoluble protein aggregates positive for ubiquitin (Fig 7A). The formation of these aggregates is followed by an increase in the levels of the sEVs marker CD63 recovered in media supernatants, indicating an increase in the release of sEVs. These sEVs are enriched in ubiquitinated and oligomerized proteins (Fig 7B and 7C). Interestingly, incubation with GOx also induced the presence of STUB1 and p62 in sEVs (Fig 7B).

When ARPE-19 cells are depleted from STUB1 and further incubated with GOx there is a substantial increase in the release of sEVs (Fig 7D). Rather unexpectedly, the expression of either one of the STUB1-DN mutants abrogated the release of sEVs observed following incubation with GOx (Fig 7E).

Results presented thus far suggest that proteostasis deregulation leads to an increase in the release of exosomes loaded with ubiquitinated and oligomerized proteins. Intriguingly, the abrogation of exosome release upon GOx incubation, in cells expressing STUB1-DN mutants, implies a role for STUB1 in exosome release that is more complex than anticipated.

### STUB1 and the Heat Shock Factor 1 (HSF1) coordinate to regulate sEVs release

HSF1 is a transcription factor known to regulate the synthesis of molecular chaperones, thereby mediating cellular response to proteostasis deregulation. Some reports have shown that STUB1 is involved in the activation of HSF1 in a process that does not involve the ubiquitination of HSF1 but rather the binding of STUB1 to HSF1 and its translocation into the nucleus [29, 30]. Taking our data into consideration, we hypothesized that the expression of STUB1-DN mutants might be activating HSF1.

**Fig 8:**
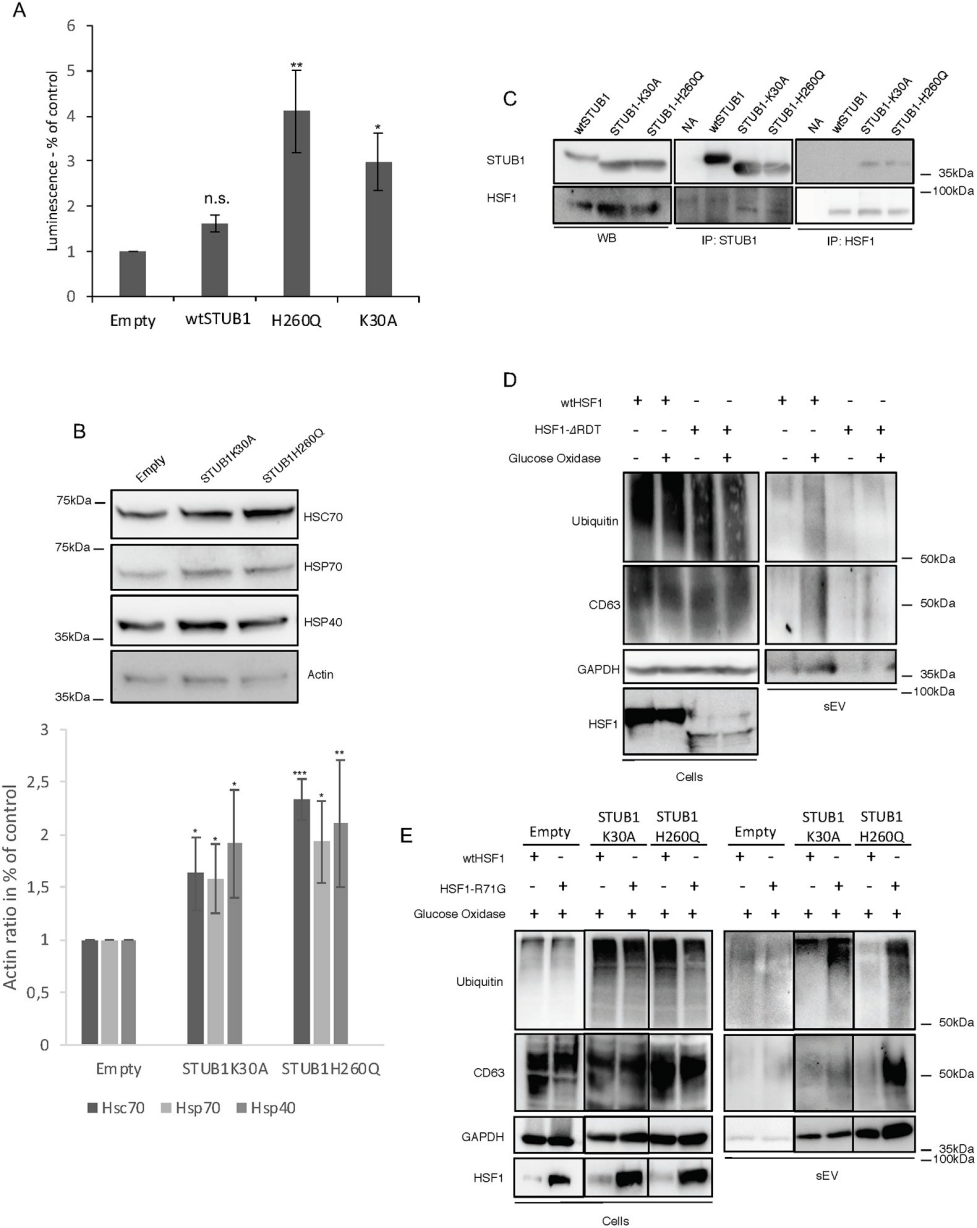
Expression of STUB1-DN increases HSF1 activity. Activation of HSF1 inhibits GOx induced sEVs secretion. ARPE-19 cells were transduced using lentiviral particles containing vectors either for the expression of wtSTUB1, STUB1K30A and STUB1H260Q or with adenoviral particles containing shRNA against STUB1. Control cells were transduced with an empty vector. A) Cells were transfected with a plasmid for the expression of Luciferase under the control of an HSF1 promoter (HSE sequence). STUB1-DN mutants increase the expression of Luciferase. B) SDS-PAGE of extracts from cells expressing STUB1-DN incubated with antibodies raised against HSC70, HSP70, HSP40 and actin. Cells expressing the STUB1-DN mutant show an increase in the levels of molecular chaperones. C) Immunoprecipitation experiments using antibodies raised against V5 (wtSTUB1), myc (mutant STUB1) and HSF1 indicate that STUB1-DN mutants show increased interaction with HSF1, when compared with wtSTUB1. (D,E) Cells were transfected with plasmids for the expression of either wtHSF1, HSF1-*Δ*RDT or HSF1-R71G. Cells were further maintained in the presence or absence or 40 mU of GOx for 12h. SDS-PAGE of cell extracts or sEVs isolated by sequential centrifugation of cell culture media, were blotted with antibodies against ubiquitin, CD63, GAPDH or HSF1. (D) Expression of a constitutively active HSF1 (HSF1-*Δ*RDT) leads to the inhibition of sEVs release in the presence of GOx. (E) Expression of a dominant negative HSF1 (HSF1-R71G) rescues sEVs release upon GOx incubation. All samples were analyzed under the same experimental conditions. The results represent the mean ±SD of N = 3 independent experiments (n.s. nonsignificant; *p < 0.05; **p < 0.01; ***p < 0.001).

To monitor the activity of HSF1 in cells expressing STUB1-DN mutants, cells were transduced with the reporter gene Luciferase, under the control of the Heat Shock Element (HSE), HSF1 DNA binding sequence (Fig 8A). Cells expressing STUB1-DN mutants show higher luciferase activity. To further confirm this increase of HSF1 activity, cell extracts were probed with antibodies directed against proteins under the control of HSF1, such as the molecular chaperones HSC70, HSP70 and HSP40 (Fig 8B). Expression of STUB1-DN mutants increased the protein levels of molecular chaperones. Consistently, immunoprecipitation experiments show an increase in the co-precipitation of the STUB1-DN mutants with HSF1, when compared to wtSTUB1 (Fig 8C). These results indicate that STUB1-DN mutants expression induces the activation of HSF1.

To monitor the impact of HSF1 activation in sEVs secretion, ARPE-19 cells are transfected with two different HSF1 mutants, a constitutively active mutant (HSF1-*Δ*RDT) and a DN mutant (HSF1-R71G) [49]. Data shows that the constitutively active HSF1-*Δ*RDT mutant is able to inhibit the release of sEVs after the incubation of cells with GOx (Fig 8D). On the other hand, expression of the dominant-negative HSF1-R71G, in cells also expressing the STUB1-DN mutants, is sufficient to restore GOx-induced sEVs release (Fig 8E).

Overall the data indicates that the activation of HSF1, mediated by STUB1-DN mutants, can inhibit sEVs secretion induced by oxidative stress.

## Discussion

In this manuscript we propose that exosomes are the extracellular component of a complex network that regulate cell proteostasis. Our data is consistent with a model in which undegraded proteins are loaded into the nascent intraluminal vesicles of MVEs, for subsequent secretion into the extracellular space upon MVE fusion with the plasma membrane. This mechanism is activated upon STUB1 inhibition, as well as, by oxidative stress and is likely to contribute to the extracellular deposition of proteotoxic material that is a hallmark of ageing and age-related diseases.

We showed that STUB1 inactivation leads to the accumulation of proteasomal substrates and to the formation of intracellular insoluble protein aggregates. These aggregates are positive for ubiquitin, p62 and STUB1. STUB1 is known to prevent age-related accumulation of protein aggregates [25, 28, 50]. Upon ageing STUB1 is increasingly recruited to, and trapped in, protein aggregates, leading to its loss of function [50]. Our data indicates that STUB1 inactivation also leads to an increase in the release of exosomes loaded with ubiquitinated and oligomerized proteins. We further observed that STUB1 inactivation leads to an increase in the recruitment of ubiquitinated proteins into intracellular vesicles, most likely MVEs. These observations are consistent with a model in which STUB1 inactivation acts as a trigger for the uptake of undegraded, and/or oligomerized, proteins during MVE biogenesis leading to their subsequent release in exosomes. This exosome-mediated secretion might provide a means to reduce toxicity associated with the intracellular accumulation of aggregated proteins. In further support of this hypothesis, is the observation that the activity of STUB1 is diminished in aged organisms [50, 51], presumably contributing to the increased release of exosomes and the formation of extracellular aggregates in many age-related diseases.

ROS are known to cause protein damage, leading to protein unfolding [1]. An initial response to protein unfolding involves protein refolding, as well as, Ubiquitin-Proteasome System (UPS) and Autophagic/Lysosomal mediated degradation. However ageing is also associated to a progressive impairment of proteolysis [1], further contributing to deregulation of proteostasis. Thus, it is tempting to hypothesize that loading and elimination of undegraded, or otherwise toxic proteins, via exosomes provides an additional means to restore intracellular proteostasis. Consistently, we and others [47] have shown that oxidative stress is a stimulus for the release of exosomes. These exosomes are loaded not only with ubiquitinated/oligomerized proteins, but also with the protein aggregates marker p62 and STUB1.

Interestingly, inactivation of the ubiquitin ligase activity of STUB1 increases cells and organisms sensitivity to oxidative stress [50]. Our data indicates that, while the depletion of STUB1 led to an increase in exosome release after GOx incubation, the expression of STUB1-DN mutants did the opposite. This observation may, in part, be explained by the fact that STUB1 is known to activate HSF1, a transcription factor that regulates the synthesis of molecular chaperones, therefore modulating the cell response to proteotoxic stress [29, 30]. Cells expressing STUB1-DN mutants showed higher HSF1 activity. In fact, cells expressing STUB1-DN mutants showed higher HSF1 activity than cells expressing wtSTUB1. Previous reports showed that while wtSTUB1 and a ubiquitination defective mutant (D253N/R254G) STUB1 increased HSF1 transcriptional activity, the STUB1K30A mutant failed to do so [29]. In contrast with this initial report, our data shows that STUB1K30A is also able to activate HSF1 [9], whereas wtSTUB1 is not. While we cannot fully explain these inconsistencies between our data and the data from previous works, it is likely that the use of different cell models may account for, at least part, of the discrepancies in the results. In addition, we suggest that cells expressing STUB1-DN fail to respond to oxidative stress by increasing the release of exosomes due to the association of STUB1 with HSF1. Thus, it is reasonable to hypothesize that STUB1-DN activation of HSF1 will result in an increase in the levels of molecular chaperones that help cells to cope with situations of oxidative stress. The resulting effect is the abrogation of the GOx induced release of exosomes. Consistently, the expression of an HSF1-DN mutant restored the levels of exosome secretion upon oxidative stress, while a constitutively active HSF1 inhibited the release of exosomes in the presence of GOx.

Overall our data indicates that the depletion of STUB1 might be a more accurate approach to mimic age-dependent STUB1 loss of function than the expression of the STUB1-DN mutants, particularly in the context of protein clearance through exosomes. Our results are consistent with a model in which the age-dependent loss of function of STUB1 [50] may lead to a deficient activation of HSF1. In response to this progressive decrease of HSF1 activity during ageing, cells would act by disposing of the damaged proteins via exosomes.

Previous reports suggested models where inactivation of STUB1 upon ageing leads to a decrease in the degradation of protein substrates and to the intracellular accumulation of toxic protein oligomers and aggregates [50]. We now suggest that following age-related inactivation of STUB1, cells reroute undegraded proteins to exosomes as an alternative mechanism for clearance of proteotoxic material. While we anticipate that this mechanism may be beneficial for cells under stress, we cannot exclude the possibility that the secretion of toxic proteins via exosomes, over a prolonged period of time, can prove harmful and even pathogenic to the neighboring cells or affected tissues [10–12]. Therefore, a thorough examination of the molecular mechanisms that regulate the loading of cytosolic proteins into exosomes might lead to a better understanding and prevention of age-related diseases that involve the intracellular accumulation or extracellular deposition of proteotoxic material.

## Supporting information

S1 Fig Colocalization of ubiquitin and P62 upon STUB1-DN mutants expression. ARPE-19 cells were transduced using lentiviral particles containing vectors for the expression of either STUB1K30A or H260Q. Control cells were transduced with empty vector. Immunofluorescence using confocal microscopy with antibodies against ubiquitin and p62 show increased formation of ubiquitin and p62 positive puncta and an increase in colocalization in cells expressing STUB1-DN mutants.
(PDF)

S2 Fig sEVs purified from sucrose gradient are loaded with oligomerized proteins. (A,B) Monomers, oligomers and fibrils of APP and a-Synuclein were either separated in a SDS-PAGE or trapped in a nitrocellulose membrane by a filter trap assay. Samples were subsequently blotted with antibodies raised against APP, a-Synuclein or protein oligomers (A11) The results show that the oligomer antibody successfully recognizes oligomers from both APP and a-Synuclein. Moreover the A11 antibody fails to recognize protein fibrils as previously reported. C) ARPE-19 cells were transduced using lentiviral particles containing vectors either for the expression of the STUB1K30A and STUB1H260Q mutants. Control cells were transduced with an empty vector. 10 ug of isolated sEVs were separated in a discontinuous sucrose gradient. The 8 recovered fractions were filtered through a nitrocellulose membrane and blotted with antibodies raised against CD63 and protein oligomers. Results show that the expression of STUB1-DN mutants induces the loading of oligomerized proteins in sEVs enriched fractions. All samples were analyzed under the same experimental conditions.
(PDF)

S3 Fig Lysosome inhibition is a strong stimulus for sEVs release. ARPE-19 cells were transduces using lentiviral particles containing vectors for the expression of either STUB1K30A or STUB1H260Q. Control cells were transduced with empty vector. Cells were further incubated in the presence or absence of 10uM of MG-132 and 50nM of BafA for 12h. MG-132 induces a mild increase in the release of exosomes. BafA is a potent inducer of exosome release. All samples were analyzed under the same experimental conditions.
(PDF)

S4 Fig Rab27 depletion prevents the secretion of proteasomal substrates by sEVs upon STUB1 inactivation. ARPE-19 cells were transduced using lentiviral particles containing vectors for the expression of either STUB1K30A or STUB1H260Q, with adenoviral particles containing shRNA against STUB1 or with adenoviral particles containing miRNA against Rab27. Control cells were transduced with an empty vector. A) Particle counting using nanoparticle tracking system (NanoSight). Rab27 depletion decreases the number of sEVs, smaller than 200nm, released by ARPE-19 cells. B,C) Western blot of whole cell lysates and sEVs sample with antibodies against CD63, HIF1A, mutYH and p53. The depletion of Rab27 inhibits the secretion of expression of proteasomal substrates in released sEVs induced by STUB1 inactivation. All samples were analyzed under the same experimental conditions.
(PDF)

S5 Fig Ubiquitin colocalizes with MVEs in cells expressing STUB1-DN mutants. ARPE-19 cells were transduced using lentiviral particles containing vectors for the expression of either STUB1K30A or STUB1H260Q. Control cells were transduced with empty vector. A) Immunofluorescence using with antibodies against ubiquitin and the RhoB-PE dye for MVE labeling shows an increase in puncta positive for both ubiquitin and RhoB-PE. The results represent the mean ±SD of at least three independent experiments (n.s. nonsignificant; *p < 0.05; **p < 0.01; ***p < 0.001).
(PDF)

S6 Fig Ubiquitin colocalizes with MVEs in cells depleted for STUB1. ARPE-19 cells were transduced using adenoviral particles containing shRNA against STUB1. Control cells were transduced with empty vector. A) Immunofluorescence using confocal microscopy with antibodies against STUB1, ubiquitin and the RhoB-PE dye for MVE labeling shows an increase in puncta positive for ubiquitin and RhoB-PE. B) Quantification of size and number of vesicles labelled with RhoB-PE dye shows an increase in the frequency of larger vesicles in STUB1 depleted cells.
(PDF)
