# Supplementary figures and images for: Exosomes and STUB1/CHIP cooperate to maintain intracellular proteostasis
Source: PLoS One. 2019 Oct 15;14(10):e0223790. doi: 10.1371/journal.pone.0223790 (PMC6794069; doi:10.1371/journal.pone.0223790)

Supplementary Figure 1

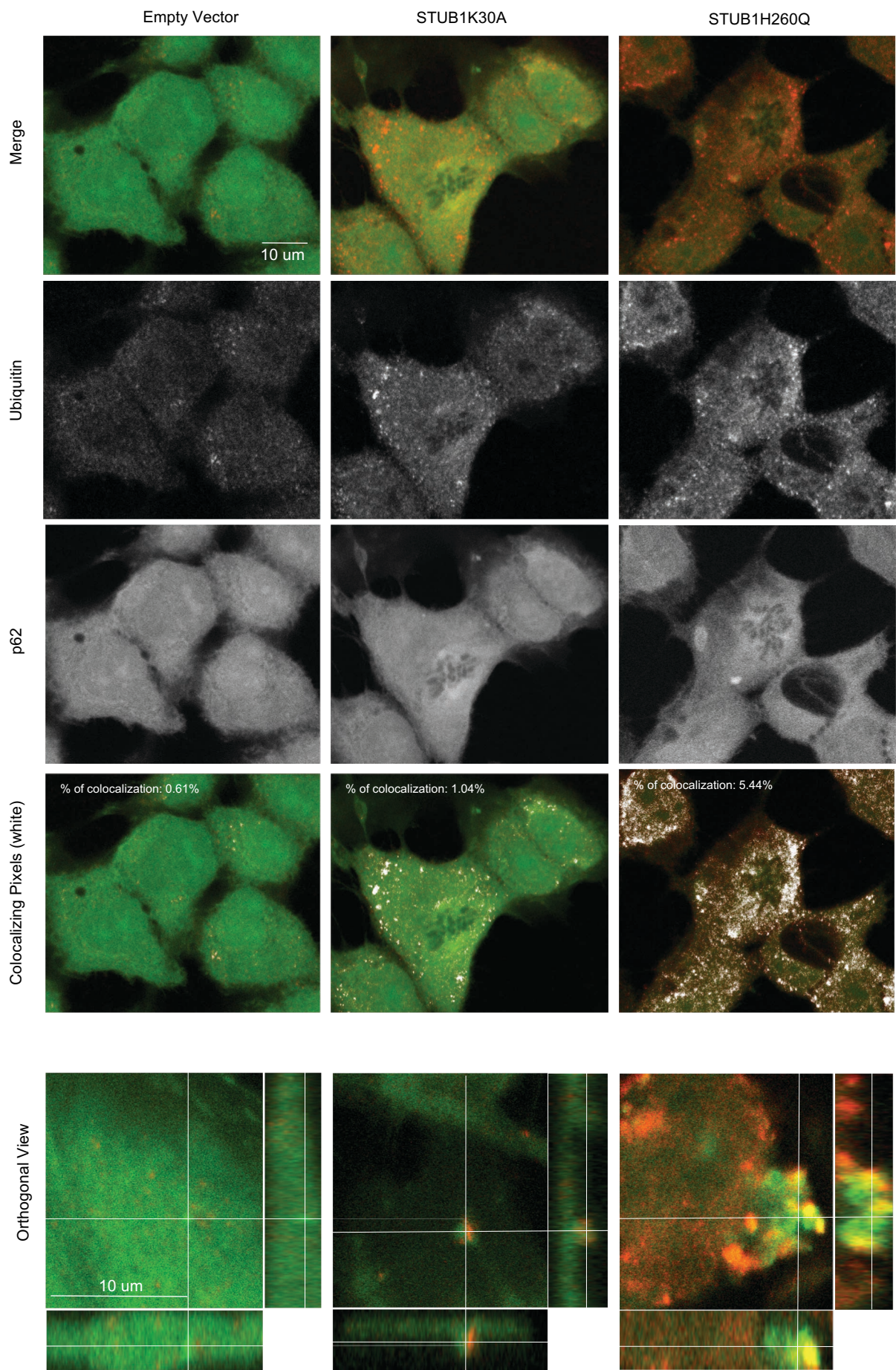

Supplement: S1 Fig — ARPE-19 cells were transduced using lentiviral particles containing vectors for the expression of either STUB1K30A or H260Q. Control cells were transduced with empty vector. Immunofluorescence using confocal microscopy with antibodies against ubiquitin and p62 show increased formation of ubiquitin and p62 positive puncta and an increase in colocalization in cells expressing STUB1-DN mutants. (PDF) [file pone.0223790.s001.pdf]

Supplementary Figure 3

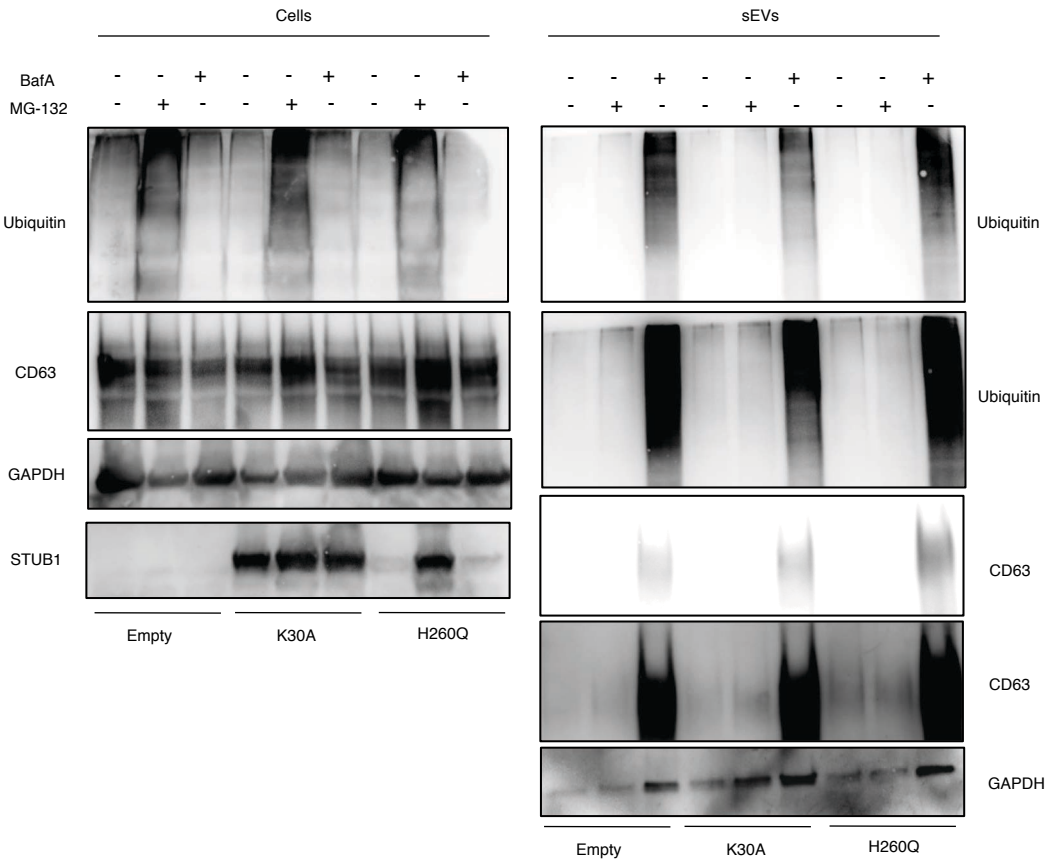

Supplement: S3 Fig — ARPE-19 cells were transduces using lentiviral particles containing vectors for the expression of either STUB1K30A or STUB1H260Q. Control cells were transduced with empty vector. Cells were further incubated in the presence or absence of 10uM of MG-132 and 50nM of BafA for 12h. MG-132 induces a mild increase in the release of exosomes. BafA is a potent inducer of exosome release. All samples were analyzed under the same experimental conditions. (PDF) [file pone.0223790.s003.pdf]

Supplementary Figure 4

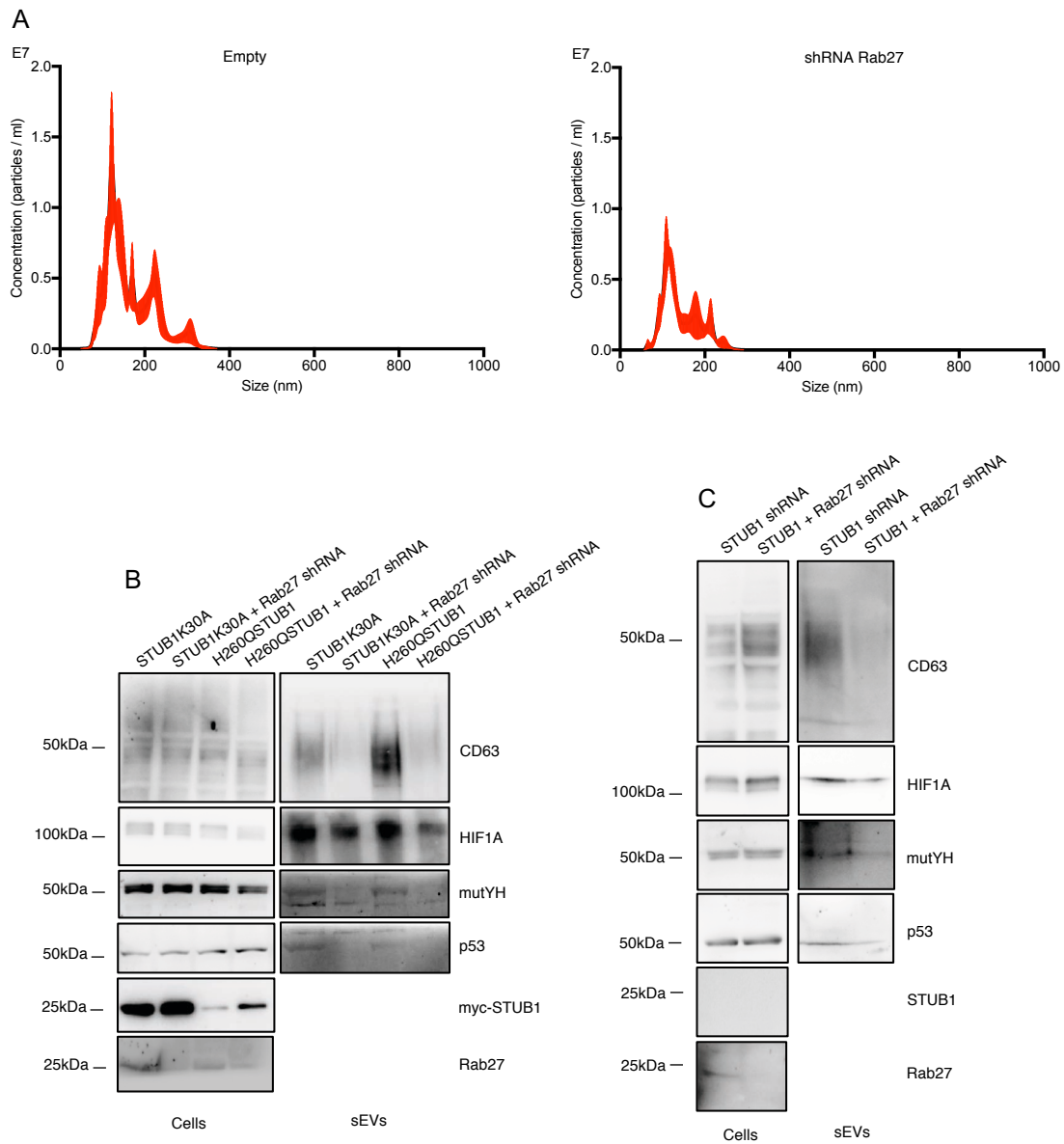

Supplement: S4 Fig — ARPE-19 cells were transduced using lentiviral particles containing vectors for the expression of either STUB1K30A or STUB1H260Q, with adenoviral particles containing shRNA against STUB1 or with adenoviral particles containing miRNA against Rab27. Control cells were transduced with an empty vector. A) Particle counting using nanoparticle tracking system (NanoSight). Rab27 depletion decreases the number of sEVs, smaller than 200nm, released by ARPE-19 cells. B,C) Western blot of whole cell lysates and sEVs sample with antibodies against CD63, HIF1A, mutYH and p53. The depletion of Rab27 inhibits the secretion of expression of proteasomal substrates in released sEVs induced by STUB1 inactivation. All samples were analyzed under the same experimental conditions. (PDF) [file pone.0223790.s004.pdf]

Supplementary Figure 5

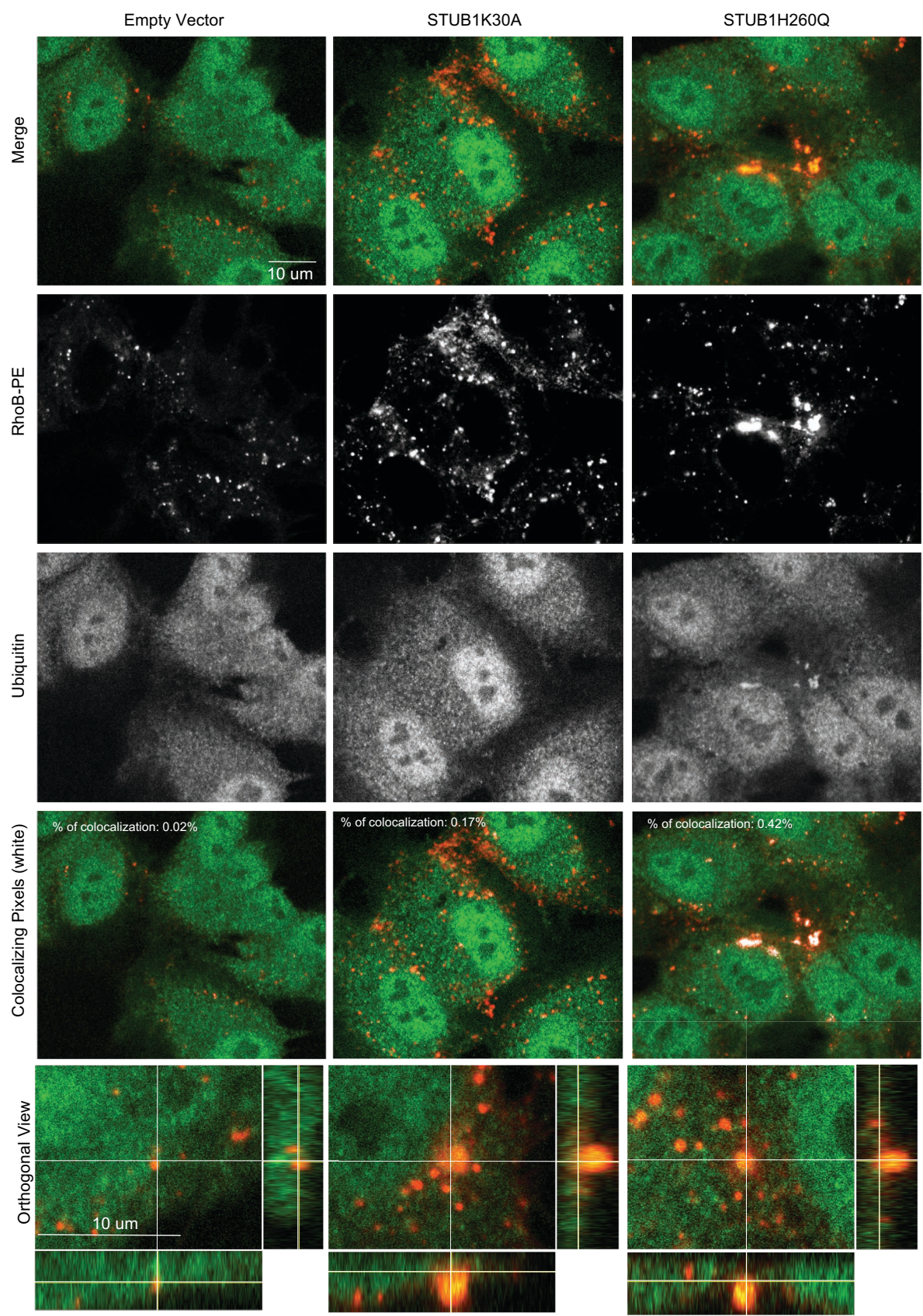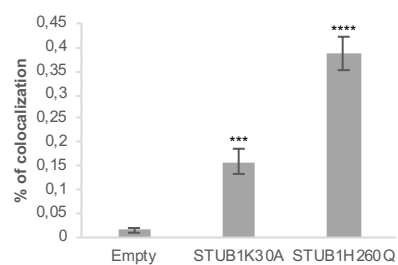

Supplement: S5 Fig — ARPE-19 cells were transduced using lentiviral particles containing vectors for the expression of either STUB1K30A or STUB1H260Q. Control cells were transduced with empty vector. A) Immunofluorescence using with antibodies against ubiquitin and the RhoB-PE dye for MVE labeling shows an increase in puncta positive for both ubiquitin and RhoB-PE. The results represent the mean ±SD of at least three independent experiments (n.s. nonsignificant; *p < 0.05; **p < 0.01; ***p < 0.001). (PDF) [file pone.0223790.s005.pdf]

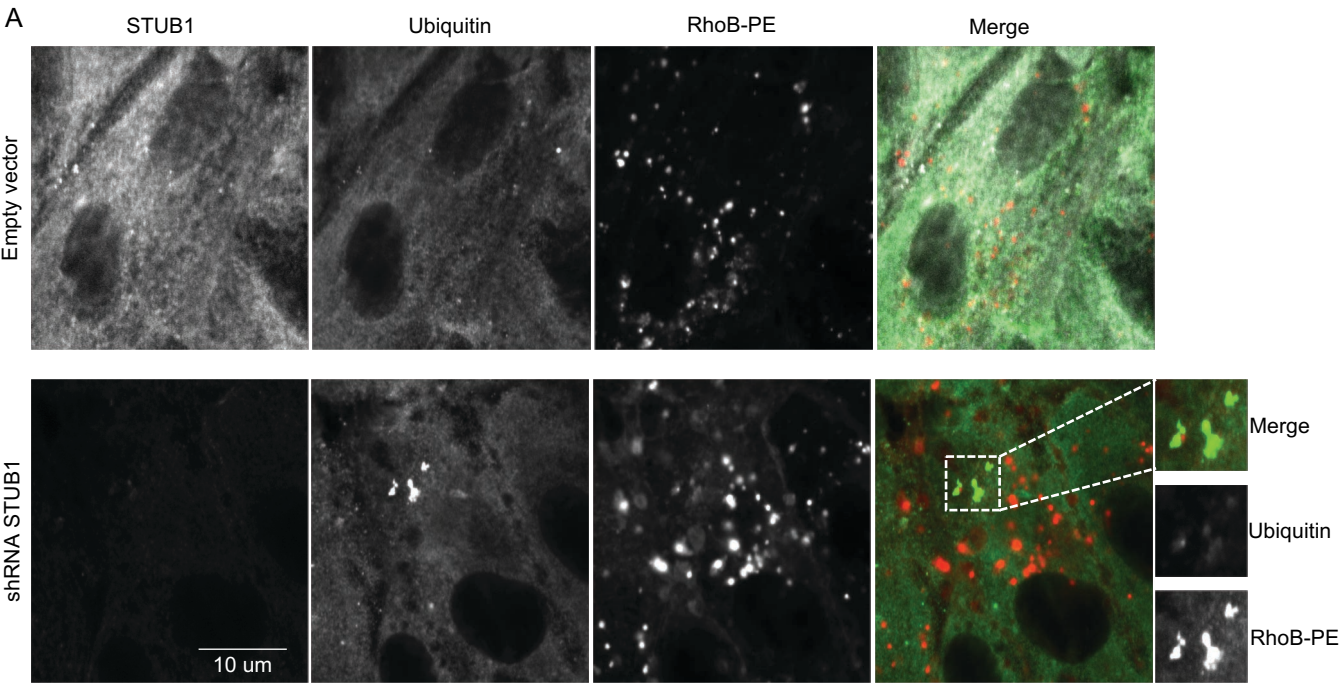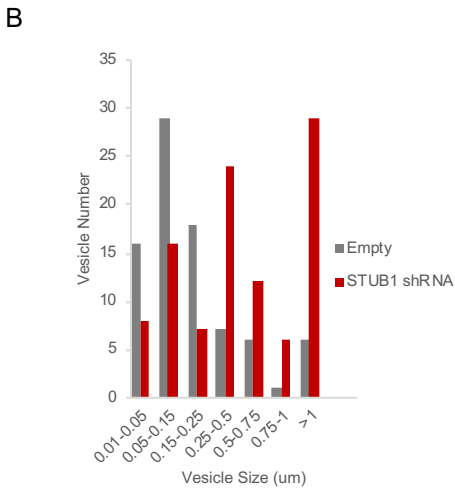

Supplement: S6 Fig — ARPE-19 cells were transduced using adenoviral particles containing shRNA against STUB1. Control cells were transduced with empty vector. A) Immunofluorescence using confocal microscopy with antibodies against STUB1, ubiquitin and the RhoB-PE dye for MVE labeling shows an increase in puncta positive for ubiquitin and RhoB-PE. B) Quantification of size and number of vesicles labelled with RhoB-PE dye shows an increase in the frequency of larger vesicles in STUB1 depleted cells. (PDF) [file pone.0223790.s006.pdf]
