# Supplementary material for: Exosomes and STUB1/CHIP cooperate to maintain intracellular proteostasis
Source: PLoS One. 2019 Oct 15;14(10):e0223790. doi: 10.1371/journal.pone.0223790 (PMC6794069; doi:10.1371/journal.pone.0223790)

Supplementary Figure 2

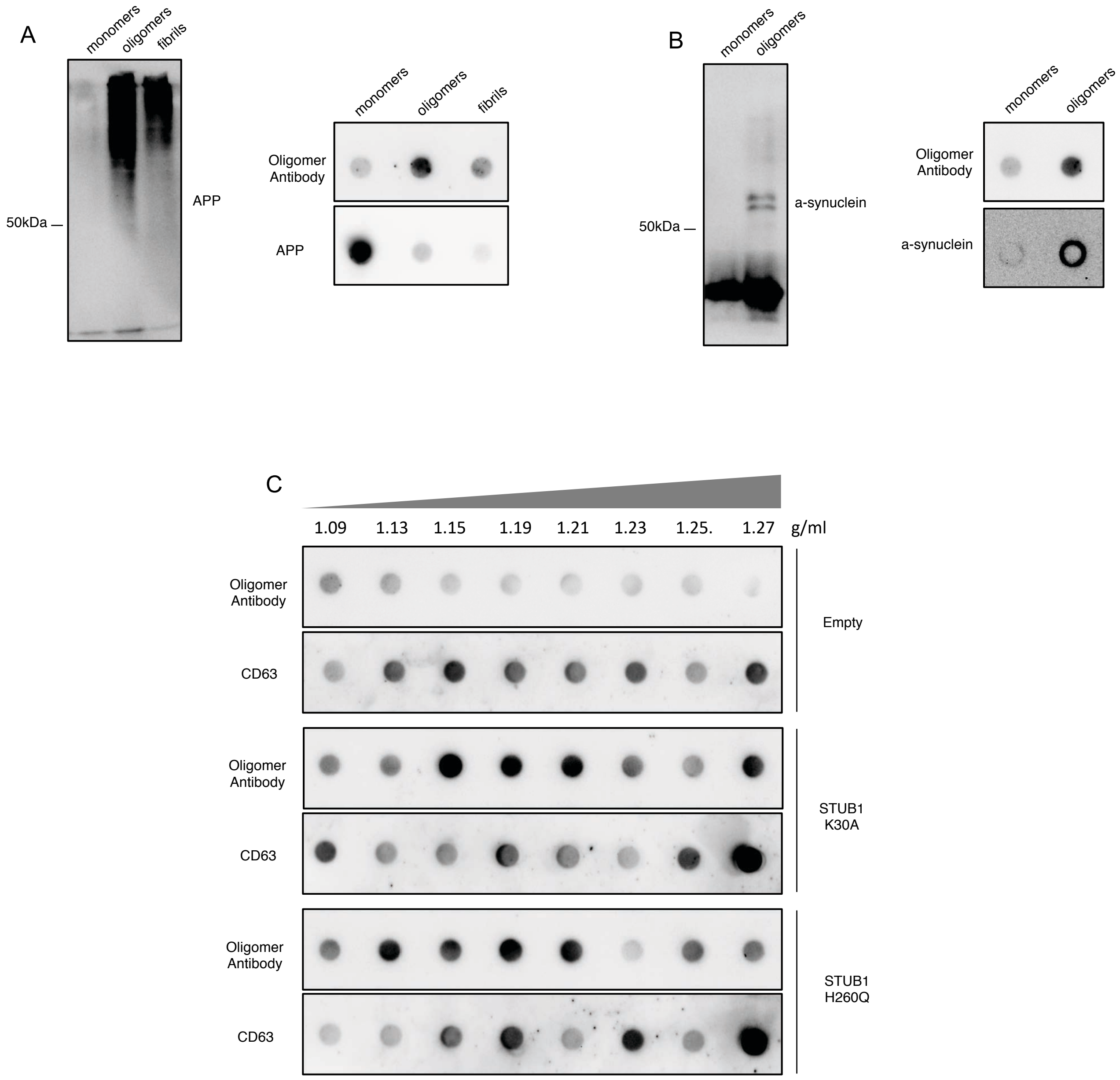

Supplement: S2 Fig — (A,B) Monomers, oligomers and fibrils of APP and a-Synuclein were either separated in a SDS-PAGE or trapped in a nitrocellulose membrane by a filter trap assay. Samples were subsequently blotted with antibodies raised against APP, a-Synuclein or protein oligomers (A11) The results show that the oligomer antibody successfully recognizes oligomers from both APP and a-Synuclein. Moreover the A11 antibody fails to recognize protein fibrils as previously reported. C) ARPE-19 cells were transduced using lentiviral particles containing vectors either for the expression of the STUB1K30A and STUB1H260Q mutants. Control cells were transduced with an empty vector. 10 ug of isolated sEVs were separated in a discontinuous sucrose gradient. The 8 recovered fractions were filtered through a nitrocellulose membrane and blotted with antibodies raised against CD63 and protein oligomers. Results show that the expression of STUB1-DN mutants induces the loading of oligomerized proteins in sEVs enriched fractions. All samples were analyzed under the same experimental conditions. (PDF) [file pone.0223790.s002.pdf]
